# Patterns and trends of cancer incidence in children and adolescents in China, 2011–2015: A population‐based cancer registry study

**DOI:** 10.1002/cam4.4014

**Published:** 2021-06-02

**Authors:** Kexin Sun, Rongshou Zheng, Siwei Zhang, Hongmei Zeng, Shaoming Wang, Ru Chen, Wenqiang Wei, Jie He

**Affiliations:** ^1^ National Central Cancer Registry National Cancer Center/National Clinical Research Center for Cancer/Cancer Hospital Chinese Academy of Medical Sciences and Peking Union Medical College Beijing China; ^2^ Department of Thoracic Surgery National Cancer Center/National Clinical Research Center for Cancer/Cancer Hospital Chinese Academy of Medical Sciences and Peking Union Medical College Beijing China

**Keywords:** adolescent, child, China, incidence, neoplasms

## Abstract

**Background:**

Cancer is a major concern for children and adolescents worldwide. This study aims to report on cancer incidence patterns at age 0–19 years in 2011–2015 and their trends in 2000–2015.

**Methods:**

We collected data on malignancies in population of 0–19 years submitted by high‐quality population‐based cancer registries in China. Age‐standardized rates by world standard population (WSR) and annual percent change (APC) were calculated.

**Results:**

In total, 215 cancer registries from 30 provinces contributed datasets during 2011–2015. Twenty‐two registries provided continuous data for trend analysis from 2000 to 2015. In total 16,954 malignancies occurred in 177,416,582 person‐years. WSRs were 93.32 and 96.03 per million person‐years in children aged 0–14 and 0–19 years. Incidence rates were higher in boys than in girls and were higher in urban area than in rural area. In children aged 0–14 years, the top three common diagnostic groups were leukemia, central nervous system (CNS) tumors, and lymphomas in both sexes. In adolescents aged 15–19 years, the top three common diagnostic groups were leukemia, epithelial tumors and melanoma, and CNS tumors in boys and epithelial tumors and melanoma, leukemia, and germ cell and gonadal tumors in girls. WSRs for cancers in 0–19 years of age increased significantly in boys from 2000 to 2005 (APC = 5.3%, 95% CI: 2.3%–8.3%) and in girls from 2000 to 2015 (APC = 1.2%, 95% CI: 0.1%–2.4%).

**Conclusions:**

Cancer incidence in children and adolescents is on the rise in China. The observed age, sex, and geographical variations in cancer incidence should be used to inform targeted prevention and control policies.

## Graphical abstract

We used qualified cancer registry data to report nationwide incidence patterns of childhood cancer in China. During 2011—2015, 16954 malignancies in total were found. Age‐standardized incidence rates were 93.32 and 96.03 per million in 0–14 and 0–19 years of age. Incidence rates were higher in boys than in girls and were higher in urban area than in rural area. Leukemia, central nervous system tumors and lymphomas were common in children of 0–14 years. Epithelial tumors and melanoma, germ cell and gonadal tumors were common in adolescents of 15–19 years. Cancer incidence increased significantly during 2000—2015.

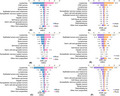

## 1 INTRODUCTION

Cancers occurring before age 20 years are rare. However, the recorded incidence of childhood cancer tended to increase with time in the past three decades.^1^ Nowadays, these neoplasms have already become major life‐threatening concerns for children and adolescents worldwide. It was reported that cancer was the 9th leading cause of childhood disease burden globally in 2017, which accounted for a substantial global disability adjusted life year (DALY) burden. Over 80% of global childhood cancer disease burden occurred in low‐income and middle‐income regions.^2^, ^3^

As one of the developing countries in the world, the disease burden of childhood cancer in China is also high. According to data of the Chinese Disease Surveillance Points System, cancer is the 8th and the 2nd cause of death for children <1 year old and 1–19 years old, respectively.^4^ We searched PubMed and China National Knowledge Infrastructure, without language restrictions, for research articles published before 25 August 2020, using the terms “pediatric or childhood or child or adolescent,” “cancer,” “incidence,” “China.” We found that the national childhood cancer profile in China was reported for the first time in 2015,^5^ which attracted great attention at home and abroad. In recent years, the diagnosis and treatment capability of childhood cancer in China has been improving. However, no updated population‐based statistics were reported. To address this issue, we are going to assess the childhood cancer incidence pattern in China using quality‐assured cancer registry datasets during 2011–2015. These findings can provide scientific evidences for the establishment of childhood cancer prevention and control plans in China.

## 2 MATERIALS AND METHODS

### 2.1 Data source and data acquisition

We reported the incidence of childhood cancer using nation‐wide population‐based cancer registry datasets. The current status of cancer registration, the collection, and quality control processes of cancer registry data in China were described in detail elsewhere.^6^, ^7^

Data submitted by cancer registries went through standard quality control process. The completeness of dataset, the consistency between variables and the population structure were checked based on the quality control criteria of International Agency for Research on Cancer/International Association of Cancer Registries (IARC/IACR).^8^, ^9^ Questionable records were fed back to the contributing registries for double check and correction. Cancer registry datasets which met quality control criteria in individual years of 2011–2015 were selected. Among these, we selected registries covering entire 5 years (2011–2015) or four successive years (2011–2014 or 2012–2015) or recent 3 years (2013–2015) as qualified registries. Incidence and population datasets of corresponding years of each qualified registry were pooled together.

Among all registries, 22 cancer registries can provide continuous surveillance data with good quality since year 2000. Datasets from 2000–2015 of these 22 registries were included for trend analysis.

Cancer registries in China coded incident cases according to the International Classification of Diseases for Oncology, Third Edition (ICD‐O‐3). The ICD‐O‐3 codes were subsequently converted to International Classification of Childhood Cancer, third edition (ICCC‐3).^10^ According to Chinese Guideline for Cancer Registration, we registered all malignancies and non‐malignant neoplasms of the central nervous system (CNS). However, non‐malignant neoplasms of the CNS were excluded because of high missing report rates. Thus we only included malignancies before age 20 years.

### 2.2 Statistical analysis

Since resulting statistics of childhood cancer were more sensitive to imprecision, we further evaluated the quality of pooled childhood cancer datasets of every cancer registry according to a set of quality control indicators. (1) Crude incidence rate (CR): Since the incidence of childhood cancer was low, drastic fluctuations of CR could be observed especially in counties with limited population. We calculated the CR of each registry. We then calculated the mean and square deviation (SD) of all CRs and considered CRs within the range of mean ± 2 × SD were acceptable. We removed registries with unacceptable CRs and did the analysis again. This process was repeated until CRs of all remaining registries were within the prescribed limits. (2) Proportion of cases registered from death certificate (proportion of death certificate only, DCO%): Cancer registries with DCO% >20% were excluded. (3) Proportion of cases diagnosed by tissue examination (proportion of microscopic verification, MV%): Cancer registries with MV% <55% were excluded. (4) Proportion of cases classified as other and unspecified malignant neoplasms subgroup of ICCC‐3 (NOS%): Cancer registries with NOS% >30% were excluded.

Datasets that have passed quality verification were pooled together. Using this pooled dataset, we calculated incidence rates (per million, 1/10^6^) as the quotient of cases and person‐years stratified by age (<1, 1–4, 5–9, 10–14, and 15–19), sex (male/female), area (urban/rural), and main diagnostic group. The person‐years of a registry were the sum of the population counts of eligible years during 2011 to 2015. Age‐specific incidence rates (ASR) were calculated for each age group. To obtain internationally comparable results, we used the Segi's population to calculate age‐standardized rates by world standard population (WSR) and their 95% CIs for 0–14 years and 0–19 years age groups. All statistical analyses were carried out by Stata (version 13.0).

We used Joinpiont Regression Program (version 4.6.0.0) to do trend analysis. We fit ASR and WSR with year using log‐transformed linear regression models. All models were restricted to a maximum of 3 joinpoints. Annual percent change (APC) and average annual percent change (AAPC) were calculated with corresponding 95% CIs. *Z* test was used to assess whether the APC or AAPC was statistically different from zero.

## 3 RESULTS

### 3.1 Basic information of contributing cancer registries

**FIGURE 1:**
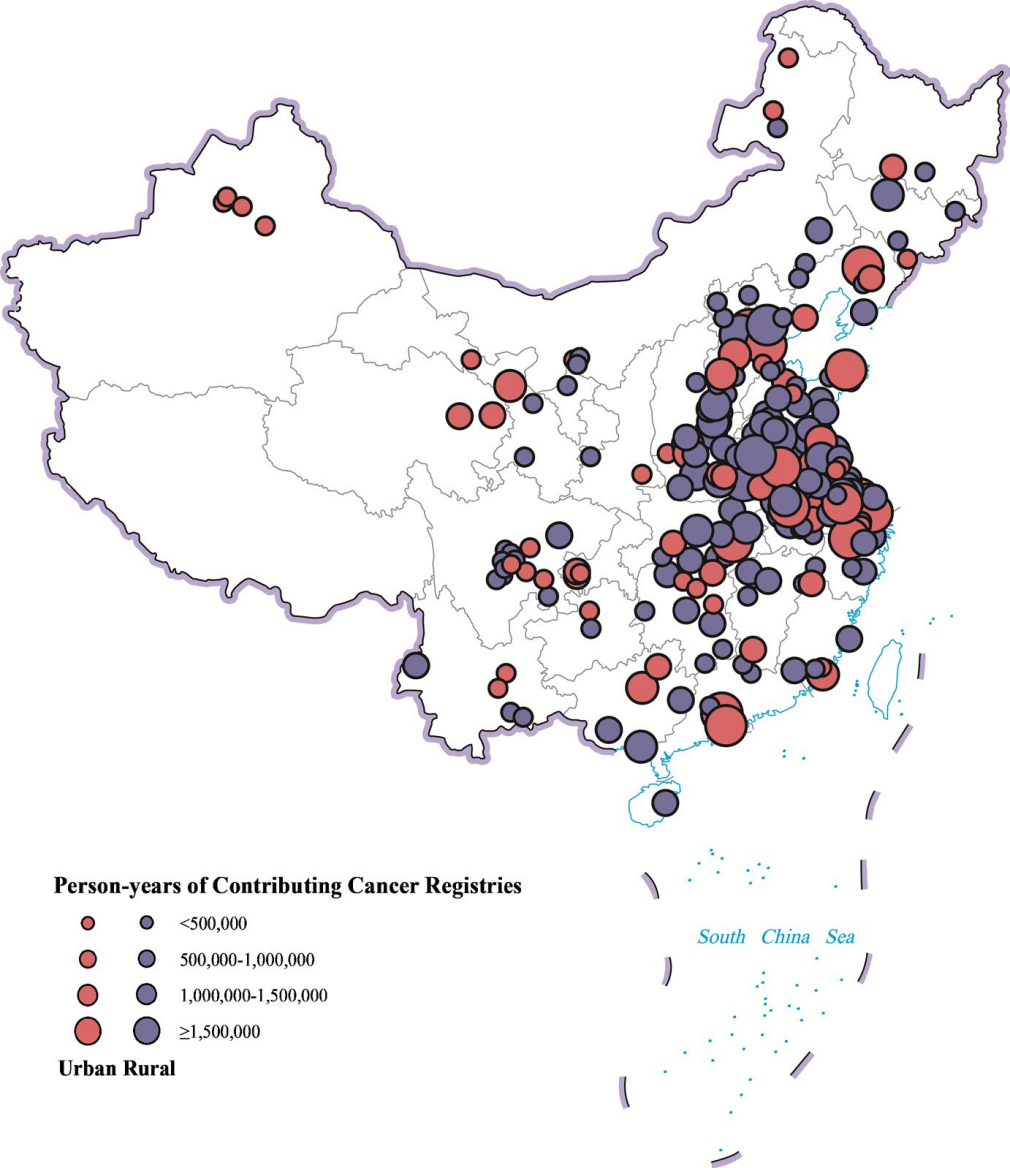
Regions covered by the 215 contributing cancer registries of childhood cancer data in China, 2011–2015, by area and person‐years

Data submitted by 177, 193, 255, 339, and 388 cancer registries met quality control criteria in 2011, 2012, 2013, 2014, and 2015, respectively. One hundred thirty‐eight registries can provide data covering entire 5 years, 30 registries can provide data covering 4 successive years (2011–2014: 4 registries; 2012–2015: 26 registries), 63 registries can provide data covering recent 3 years. Among these 231 cancer registries, 1 registry with unacceptable CR, 5 registries with unacceptable DCO%, 6 registries with unacceptable MV%, and 4 registries with unacceptable NOS % were deleted. At last, datasets of 215 cancer registries from 30 provinces (no data available from Tibet) were included and pooled together (Figure 1). 57, 29, and 129 registries contributed data of 3 years, 4 years, and 5 years, respectively. No significant differences of DCO% and MV% between 3 kinds of registries were found. MV% of CNS tumors was relatively low compared to other main diagnostic groups (Table 1).

**TABLE 1:** Data quality indicators in populations of 0–14 years and 0–19 years, by main diagnostic group

Main diagnostic groups	DCO (%)		MV (%)	
	0–14	0–19	0–14	0–19
Leukemia	0.73	0.81	97.35	97.32
Lymphomas	0.65	0.58	96.50	96.12
CNS tumors	3.99	3.23	49.97	50.30
Sympathetic nervous system tumors	0.24	0.24	96.35	96.21
Retinoblastoma	0	0	94.04	94.04
Renal tumors	0.85	0.75	78.59	77.06
Hepatic tumors	1.69	1.80	68.58	63.15
Bone tumors	1.59	1.43	69.56	71.51
Soft tissue sarcomas	0.31	0.18	97.85	98.53
Germ cell and gonadal tumors	0.52	0.26	90.39	88.72
Epithelial tumors and melanoma	0.19	0.07	98.70	99.28
Other And unspecified	6.65	5.33	34.12	38.75

Abbreviations: CNS, central nervous system; DCO%, proportion of death certificate only; MV%, proportion of microscopic verification.

Datasets of 22 registries met quality control criteria. Together they covered 44.4 million populations. The locations of 22 registries with successive data from 2000 to 2015 were reported in detail elsewhere.^6^

### 3.2 Incidence rates of all cancer types

Overall, 16,954 malignancies occurred in 177,416,582 person‐years during 2011–2015. All registries covered 12.0% of the national population before age 20 years. WSRs were 93.32 and 96.03 per million person‐years in children aged 0–14 years and 0–19 years. Incidence rates were higher in boys (101.69 per million) than in girls (89.59 per million). Incidence sex ratios ranged from 1.14 to 1.25 in different age groups between 0 and 14 years. But the ASRs in both sexes were almost equal in 15–19 years age group. Incidence rates were higher in urban area (102.72 per million) than in rural area (90.45 per million) (Table 2).

**TABLE 2:** Numbers of cancer cases, person‐years, and incidence rates in China, 2011–2015, by age group and area

Item	Total			Boys			Girls		
	Case	Person‐years (millions)	Incidence per million	Case	Person‐years (millions)	Incidence per million	Case	Person‐years (millions)	Incidence per million
Age group, WSR (95% CI)									
0–14	11,656	127.13	93.32 (91.61, 95.02)	6685	67.66	100.55 (98.13, 102.97)	4971	59.47	85.09 (82.72, 87.47)
0–19	16,954	177.42	96.03 (94.56, 97.49)	9461	93.94	101.69 (99.62, 103.77)	7493	83.48	89.59 (87.52, 91.65)
Age group, ASR									
<1	1023	8.33	122.81	578	4.41	131.07	445	3.92	113.52
1–4	4085	35.25	115.89	2336	18.8	124.26	1749	16.45	106.32
5–9	3126	41.95	74.52	1834	22.27	82.35	1292	19.68	65.65
10–14	3422	41.60	82.26	1937	22.18	87.33	1485	19.42	76.47
15–19	5298	50.29	105.35	2776	26.28	105.63	2522	24.01	105.04
Area, WSR (95% CI)									
Urban	8063	78.74	102.72 (100.45, 104.99)	4391	41.08	108.02 (104.79, 111.25)	3672	37.66	96.82 (93.64, 100.00)
Rural	8891	98.68	90.45 (88.54, 92.36)	5070	52.86	96.49 (93.79, 99.18)	3821	45.82	83.45 (80.76, 86.14)

Abbreviations: ASR, Age‐specific rate; WSR, Age‐standardized rate by world standard population.

### 3.3 Incidence rates by main diagnostic groups

**FIGURE 2:**
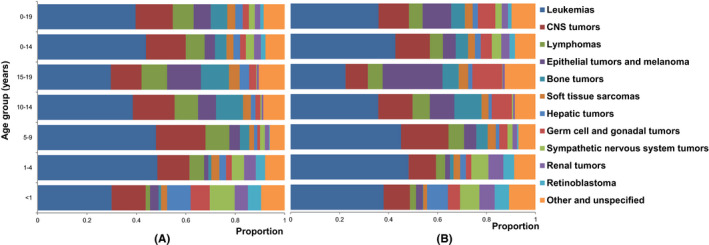
Proportional distribution of main diagnostic groups of childhood cancer, 2011–2015, by age group. (A) Boys; (B) Girls. CNS, Central nervous system

**FIGURE 3:**
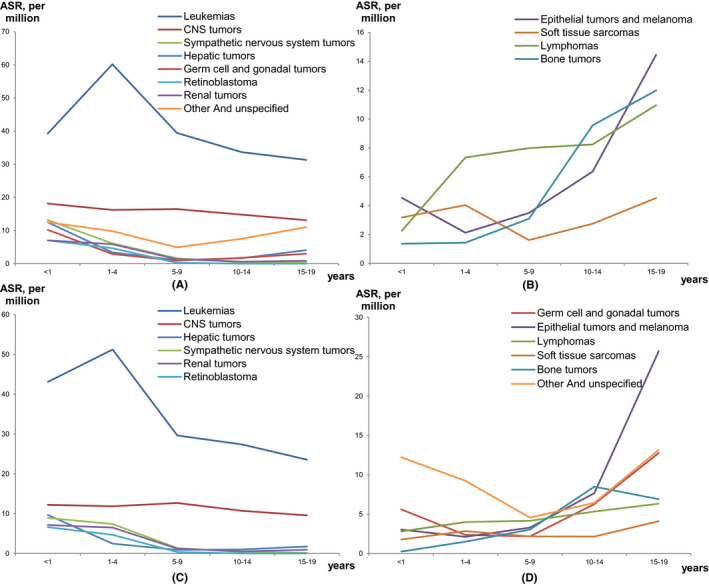
Age‐specific incidence rates of main diagnostic groups of childhood cancer, 2011–2015, by sex. (A) Cancers with downward trends across age groups in boys; (B) Cancers with upward trends across age groups in boys; (C) Cancers with downward trends across age groups in girls; (D) Cancers with upward trends across age groups in girls. ASR, Age‐specific rate; CNS, Central nervous system

The most common diagnostic group was leukemia, representing 39.7% and 35.9% of all cases in boys and girls (Figure 2). The WSRs were 41.30 and 33.76 per million in boys and girls. It was the top one diagnostic group in all age groups in boys and age groups between 0–14 years in girls. ASR of leukemia reached the highest in 1–4 years and decreased with age afterwards (Table 3; Figure 3).

**TABLE 3:** Numbers of cancer cases and age‐specific incidence rates in China, 2011–2015, by sex and main diagnostic group

Sex	Main diagnostic group	<1		1–4		5–9		10–14		15–19		0–14		0–19	
		*N*	ASR	*N*	ASR	*N*	ASR	*N*	ASR	*N*	ASR	*N*	WSR	*N*	WSR
Boys	All	578	131.07	2336	124.26	1834	82.35	1937	87.33	2776	105.63	6685	100.55	9461	101.69
	Leukemia	173	39.23	1132	60.21	879	39.47	747	33.67	823	31.31	2931	44.19	3754	41.30
	Lymphomas	10	2.27	138	7.34	178	7.99	183	8.25	288	10.96	509	7.42	797	8.22
	CNS tumors	80	18.14	305	16.22	367	16.48	328	14.79	345	13.13	1080	16.04	1425	15.38
	Sympathetic nervous system tumors	58	13.15	115	6.12	36	1.62	10	0.45	7	0.27	219	3.57	226	2.82
	Retinoblastoma	31	7.03	88	4.68	6	0.27	0	0	0	0	125	2.08	125	1.61
	Renal tumors	31	7.03	111	5.90	31	1.39	13	0.59	23	0.88	186	2.99	209	2.52
	Hepatic tumors	55	12.47	64	3.40	24	1.08	36	1.62	107	4.07	179	2.84	286	3.12
	Bone tumors	6	1.36	27	1.44	69	3.10	212	9.56	315	11.99	314	4.32	629	6.05
	Soft tissue sarcomas	14	3.17	76	4.04	36	1.62	61	2.75	119	4.53	187	2.82	306	3.20
	Germ cell and gonadal tumors	45	10.20	55	2.93	20	0.90	39	1.76	79	3.01	159	2.50	238	2.61
	Epithelial tumors and melanoma	20	4.54	40	2.13	78	3.50	141	6.36	380	14.46	279	3.99	659	6.34
	Other And unspecified	55	12.47	185	9.84	110	4.94	167	7.53	290	11.03	517	7.79	807	8.52
Girls	All	445	113.52	1749	106.32	1292	65.65	1485	76.47	2522	105.04	4971	85.09	7493	89.59
	Leukemia	169	43.11	843	51.25	583	29.63	532	27.39	566	23.58	2127	36.72	2693	33.76
	Lymphomas	11	2.81	66	4.01	82	4.17	104	5.35	152	6.33	263	4.36	415	4.80
	CNS tumors	48	12.24	195	11.86	250	12.70	208	10.71	230	9.58	701	11.83	931	11.32
	Sympathetic nervous system tumors	35	8.93	122	7.42	27	1.37	8	0.41	4	0.17	192	3.55	196	2.79
	Retinoblastoma	26	6.63	77	4.68	7	0.36	0	0	0	0	110	2.08	110	1.61
	Renal tumors	28	7.14	107	6.51	24	1.22	10	0.51	23	0.96	169	3.11	192	2.63
	Hepatic tumors	38	9.69	41	2.49	18	0.91	20	1.03	42	1.75	117	2.12	159	2.03
	Bone tumors	1	0.26	25	1.52	60	3.05	165	8.50	166	6.92	251	3.94	417	4.61
	Soft tissue sarcomas	7	1.79	47	2.86	43	2.19	42	2.16	99	4.12	139	2.36	238	2.75
	Germ cell and gonadal tumors	22	5.61	39	2.37	43	2.19	122	6.28	307	12.79	226	3.70	533	5.74
	Epithelial tumors and melanoma	12	3.06	35	2.13	65	3.30	149	7.67	617	25.70	261	4.19	878	9.03
	Other And unspecified	48	12.24	152	9.24	90	4.57	125	6.44	316	13.16	415	7.15	731	8.51

Abbreviations: ASR, Age‐specific rate; CNS, Central nervous system; WSR, Age‐standardized rate by world standard population.

The second common diagnostic group was CNS tumors, representing 15.1% and 12.4% of all cases in boys and girls (Figure 2). The WSRs were 15.38 and 11.32 per million in boys and girls. ASR of CNS tumors decreased with age (Table 3; Figure 3).

Lymphomas was the third and fifth common diagnostic group in boys and girls, representing 8.4% and 5.5% of all cases, respectively (Figure 2). The WSRs were 8.22 and 4.80 per million in boys and girls. Lymphomas had the largest incidence sex ratio (1.71) compared to other diagnostic groups. The ASRs of lymphomas increased with age in both sexes (Table 3; Figure 3).

WSRs of epithelial tumors and melanoma, and germ cell and gonadal tumors were higher in girls (9.03 and 5.74 per million) than in boys (6.34 and 2.61 per million). The ASRs of these two diagnostic groups tended to increase with age (Table 3; Figure 3).

Bone tumor was the fifth and sixth common diagnostic group in boys and girls, representing 6.6% and 5.6% of all cases, respectively (Figure 2). The WSRs were 6.05 and 4.61 per million in boys and girls. The ASRs of bone tumor increased with age (Table 3; Figure 3).

Soft tissue sarcomas, hepatic tumors, sympathetic nervous system tumors, renal tumors, and retinoblastoma were less common diagnostic groups, representing 12.2% and 11.9% of all cases in total in boys and girls (Figure 2). Sympathetic nervous system tumors, retinoblastoma, and renal tumors were mostly seen in children <10 years old, the ASRs of these three diagnostic groups decreased with age. ASRs of hepatic tumors showed a similar pattern in 0–9 years of age. However, ASR of hepatic tumors in 15–19 years age group started to increase. This increase was more significant in boys than in girls (Table 3; Figure 3).

**FIGURE 4:**
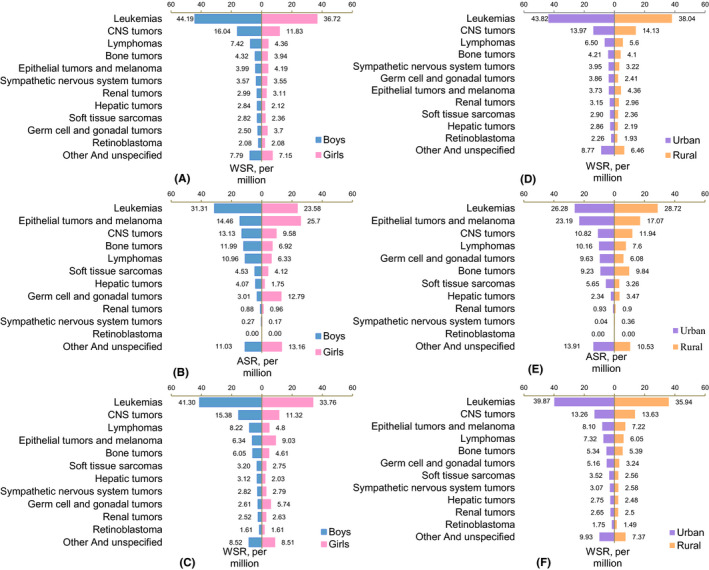
Incidence rates of main diagnostic groups of childhood cancer, 2011–2015, by sex, area and age group. (A) 0–14 years, by sex; (B) 15–19 years, by sex; (C) 0–19 years, by sex; (D) 0–14 years, by area; (E) 15–19 years, by area; (F) 0–19 years, by area. ASR, Age‐specific rate; WSR, Age‐standardized rate by world standard population; CNS, Central nervous system

The rankings of incidence rates by diagnostic group in 0–14 years and 15–19 years were different. In children aged 0–14 years, the top three common diagnostic groups were leukemia, CNS tumors, and lymphomas in both sexes. In adolescents aged 15–19 years, the top three common diagnostic groups were leukemia, epithelial tumors and melanoma, and CNS tumors in boys and epithelial tumors and melanoma, leukemia, and germ cell and gonadal tumors in girls. The rankings of incidence rates by main diagnostic group in urban area and rural area were similar (Figure 4).

A total of 1538 cases were classified as other and unspecified tumors, representing 9.1% of all cases. The proportion of other and unspecified tumors ranged from 6.0% to 12.5% in different age groups (Figure 2). Of these cases, 596 (38.8%) were diagnosed by tissue examination, 844 (54.9%) were diagnosed based on other clinical examination and 82 (5.3%) were DCO cases. Diagnostic basis of 16 (1.0%) were not clear.

### 3.4 Trends in cancer incidence from 2000 to 2015

**FIGURE 5:**
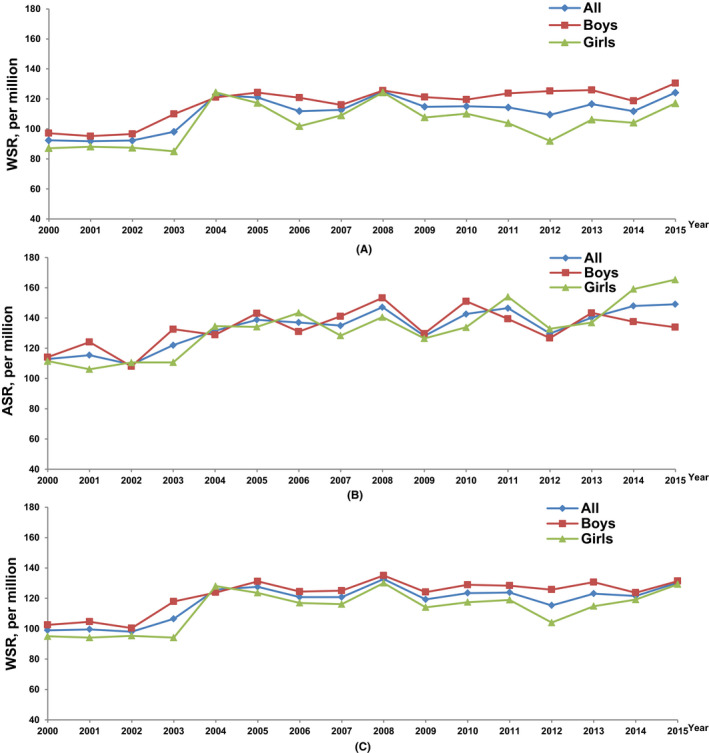
Incidence rates of childhood cancer in China, 2000–2015, by sex and age group. (A) 0–14 years, by sex; (B) 15–19 years, by sex; (C) 0–19 years, by sex. ASR, Age‐specific rate; WSR, Age‐standardized rate by world standard population

The WSR for cancers in 0–19 years of age increased significantly from 2000 to 2005 in boys (APC = 5.3%, 95% CI: 2.3%–8.3%). It then stayed stable from 2005 to 2015. The WSR for cancers in 0–19 years of age increased significantly for the entire period of 2000 to 2015 in girls (APC = 1.2%, 95% CI: 0.1%–2.4%). When analyzed by age group, the increase in boys was largely due to the increment of WSR in 0–14 years of age (2000–2004, APC = 6.8%, 95% CI: 2.6%–11.2%). And the increase in girls was largely due to the increment of ASR in 15–19 years of age (2000–2015, APC = 2.3%, 95% CI: 1.4%–3.2%) (Table 4; Figure 5).

**TABLE 4:** Incidence trends of childhood cancer for any time segments identified in joinpoint analysis, by age group and sex, 2000–2015

Age group	Sex	Trend1		Trend2		AAPC
		Years	APC (95% CI)	Years	APC (95% CI)	2000–2015
0–14	Total	2000–2004	7.7^a^ (2.2, 13.6)	2004–2015	0 (−0.9, 1)	2.0^a^ (0.6, 3.5)
	Boys	2000–2004	6.8^a^ (2.6, 11.2)	2004–2015	0.5 (−0.2, 1.2)	2.2^a^ (1.1, 3.3)
	Girls	2000–2015	0.9 (−0.6, 2.4)			0.9 (−0.6, 2.4)
15–19	Total	2000–2015	1.6^a^ (0.9, 2.3)			1.6^a^ (0.9, 2.3)
	Boys	2000–2015	0.9 (−0.1, 1.9)			0.9 (−0.1, 1.9)
	Girls	2000–2015	2.3^a^ (1.4, 3.2)			2.3^a^ (1.4, 3.2)
0–19	Total	2000–2005	5.7^a^ (1.8, 9.8)	2005–2015	−0.1 (−1.3, 1.1)	1.8^a^ (0.5, 3.1)
	Boys	2000–2005	5.3^a^ (2.3, 8.3)	2005–2015	0 (−0.9, 0.9)	1.7^a^ (0.7, 2.7)
	Girls	2000–2015	1.2^a^ (0.1, 2.4)			1.2^a^ (0.1, 2.4)

Abbreviations: AAPC, average annual percent change; APC, Annual percent change.

^a^ APC or AAPC with statistically significant trends, *p* < 0.05.

## 4 DISCUSSION

In this paper, we reported cancer incidences in children aged 0–19 years in China using data of high‐quality submitted by 215 cancer registries during 2011–2015. We found that WSRs were 93.32 and 96.03 per million person‐years in children aged 0–14 years and 0–19 years. We reported variations of incidence rates by age group, sex, area, and main diagnostic group. Cancer incidence increased significantly in children and adolescents during 2000 to 2015.

The overall incidence of childhood malignancy in China reported in this study was lower than the results reported in International incidence of childhood cancer, volume 3 (IICC‐3), and was also lower than the incidence of other countries in Asia, such as South Korea,^11^ Japan,^12^ and Thailand.^13^ IICC‐3 reported childhood cancer incidence rates of China using data from 6 registries, including Beijing, Dalian, Guangzhou, Hong Kong, Shanghai, and Zhongshan (http://iicc.iarc.fr/results/). The WSRs in China for children aged 0–14 years and 0–19 years were 131.9 and 135.0 per million person‐years, respectively,^14^ which were significantly higher than our results. Cancer registry data of Hong Kong was not included in this study. Data of Dalian was excluded because its data in 2013 did not meet quality control criteria. Beijing, Guangzhou, Shanghai, and Zhongshan all contributed cancer registry data for successive 5 years. The WSRs for children aged 0–14 years and 0–19 years were 134.5 and 137.2 per million person‐years if we only used data from these 4 registries for calculation, which was very close to the results of IICC‐3.

However, significant variations in incidence of childhood cancer were observed between registries in China, which could partly be explained by under‐diagnosis and under‐reporting. In China, medical institutions within the certain administrative region were required to routinely submit clinical records to their local population‐based cancer registry.^7^ Insufficient clinical diagnostic and therapeutic capability of childhood cancer can lead to an underestimation of its incidence,^15^ especially in undeveloped areas. To reveal the nationwide current status of childhood cancer registry, we chose to use 215 cancer registries of good quality from 30 provinces to calculate the incidence, instead of using limited registries located only in big cities.

Childhood cancer incidence is on the rise worldwide. It increased significantly by 0.54% per year in Europe.^16^ The increasing trend in incidence was also observed in USA,^17^ Canada,^18^ Australia,^19^ and Thailand.^13^ We reported the cancer incidence for children of 0–14 years in China for the first time in 2010, which was 87.1 per million. The incidence increased by 2.8% per year during 2000 to 2010.^5^ In this study, we found that the cancer incidence for children of 0–14 years had increased. But the increasing trend showed a deceleration from 2011 to 2015. However, the incidence for adolescent of 15–19 years increased by 1.6% per year during 2000 to 2015. Several factors may explain the observed increasing trend. First, the quality of cancer registry has been improved, and the under‐report rate has reduced. Second, clinical diagnostic capability and the accessibility to advanced medical diagnostic technologies have significantly enhanced in China in recent years. Third, increased exposure to lifestyle and environmental risk factors is another possible reason. Although the etiology is not clear, suggested risk factors for childhood cancer include maternal use of illicit drugs, tobacco or alcohol,^20^ genetic predisposition,^21^ in vitro fertilization,^22^ environmental contamination,^23^ pesticides,^24^ and exposure to computed tomography.^25^ The incidence trends for boys and girls varied significantly, indicating that the risk factors for population subgroups might be different. To further explore the reasons behind these temporal changes, analysis of incidence trends by diagnostic group, age group, and sex are needed to provide detailed explanations.

We found that the incidence was higher for boys than for girls and the largest difference was observed in lymphomas, which was in accordance with the global observation.^26^ Research based on data from the Surveillance, Epidemiology, and End Results (SEER) reported that Burkitt lymphoma displayed the strongest association with male sex.^27^ The underlying etiology for this sex disparity remained unclear. Higher risk factor exposure rate in boys might be one of the causes.

Most of childhood cancers are fatal without timely and standardized diagnosis and treatment. The experience of high‐income countries indicates that the 5‐year survival rates of childhood cancer can be largely improved by the amelioration of health care quality.^28^ Representative surveillance data of childhood cancer can reveal the population distribution, geographical distribution, and the changing trends of disease burden, which are crucial scientific evidences for health care resource allocation policy making. Thus, results reported in this paper are of great value for the development of childhood cancer prevention and treatment plans in China.

The WHO Global Initiative for Childhood Cancer is a new effort announced in 2018. The WHO supports governments to expand their capacities to deliver best practice in childhood cancer health care. The goal of this initiative is to increase the survival rate for childhood cancer at least by 60% by 2030.^29^ To coordinate with this effort, National Health Commission of China released a series of documents to prioritize childhood cancer. In 2018, 6 ministries released document on the treatment and management of childhood leukemia, emphasizing on the improvement of medical service level, drug supply system, and medical insurance system.^30^ National Health Commission further published childhood acute lymphoblastic leukemia and childhood acute promyelocytic leukemia clinical guidelines (2018 edition) to promote health care quality. In 2019, 5 ministries released another document on the treatment and management of childhood cancer, expanding the scope of diseases to lymphoma and other solid cancers, such as neuroblastoma, osteosarcoma, hepatoblastoma, nephroblastoma, and retinoblastoma.^31^

Since global survival rates of childhood cancer improve dramatically, the population of adult survivors continues to grow. Multidisciplinary health care approaches and special survivorship care strategies are required for this population to address morbidity caused by risk factors such as disease‐related and therapy‐related toxicities.^32^ However, researches on morbidity and mortality of childhood cancer survivors are scarce and there is no national survivorship care plan in China. In the future, we are going to carry out studies on the survival rate of childhood cancers, providing more scientific evidences for the development of risk‐based and patient‐centered care approach in China.

The main strengths of the study consist in the availability of national cancer registry data of high‐ quality during 2011–2015 and a comprehensive description of distribution of childhood cancer incidence in China. This study still has some limitations. First, 9.1% of all cases (1538 cases) were classified as other and unspecified tumors due to lack of pathologic examination or vague coding, which was higher than the proportion reported in IICC‐3.^1^ Second, exact ICDO‐3 histology codes were unclear for many cases. Thus, it was impossible to do analysis by diagnostic subgroups. Third, the missing report of childhood cancer in the registry system may lead to an underestimation of incidence. We must take this into account when interpreting the results of this paper. These limitations indicated that the quality of current cancer registry data was still insufficient for fine analysis. Quality of cancer registry data relied much on standardized clinical diagnosis and treatment, well‐developed electronic medical record system, and efficient cancer reporting and registry system. To address this issue, in the next stage we are going to put emphasis on quality improvement of cancer registration in China.

## CONFLICT OF INTEREST

The authors have declared no conflicts of interests.

## AUTHOR CONTRIBUTIONS

Kexin Sun: Conceptualization, methodology, software, formal analysis, investigation, data curation, writing ‐ original draft, visualization. Rongshou Zheng: Methodology, software, investigation, data curation. Siwei Zhang: Methodology, investigation. Hongmei Zeng and Shaoming Wang: Validation, investigation. Ru Chen: Validation, resources. Wenqiang Wei: Conceptualization, methodology, investigation, writing ‐ review & editing, supervision, project administration. Jie He: Conceptualization, supervision, project administration, funding acquisition. All authors contributed to data interpretation, critical revision of the article for important intellectual content, and approved the final submission.

## ETHICAL APPROVAL

The study is approved by the Ethics Committee of Cancer Institute and Hospital, Chinese Academy of Medical Sciences and Peking Union Medical College.

## Data Availability

The data that support the findings of this study are available on request from the corresponding author. The data are not publicly available due to privacy or ethical restrictions.
